# In Vitro Characterization of an Rgg-Family Regulator from Fish-Derived *Streptococcus parauberis* and Its Modulation by Cyclosporin A

**DOI:** 10.3390/microorganisms14040849

**Published:** 2026-04-09

**Authors:** Chuandeng Tu, Libin He, Xiangri Lin, Leyun Zheng, Dongling Zhang, Mao Lin

**Affiliations:** 1 School of Marine Biology, Xiamen Ocean Vocational College, Xiamen Key Laboratory of Intelligent Fishery, Applied Technology Engineering Center of Fujian Provincial Higher Education for Marine Resource Protection and Ecological Governance, Xiamen 361100, China; tuchuandeng@xmoc.edu.cn (C.T.); xmxsn@163.com (X.L.); 2 Fisheries Research Institute of Fujian, Xiamen 361000, China; shenhaiyuxm@163.com; 3 Fishery College, Jimei University, Xiamen 361021, China; zhangdongling@jmu.edu.cn

**Keywords:** *Streptococcus parauberis*, quorum sensing, Rgg-family regulator, EMSA, site-directed mutagenesis, cyclosporin A

## Abstract

*Streptococcus parauberis* is a major pathogen responsible for streptococcosis in both marine and freshwater fish species, causing substantial economic losses in aquaculture. The increasing prevalence of multidrug resistance has highlighted the urgent need for alternative disease control strategies. Interference with bacterial quorum sensing (QS) systems represents a promising approach. This study aimed to identify and biochemically characterize an Rgg-family transcriptional regulator and evaluate its potential as a target for quorum sensing-related regulatory interference in vitro. We hypothesized that this Rgg regulator may function as a quorum sensing-associated transcription factor capable of promoter binding and modulation by small molecules. Bioinformatic analyses were used to identify the *rgg* gene encoding an Rgg-family transcriptional regulator and predict its structural features. The gene was cloned, heterologously expressed, and purified. Promoter binding activity was examined using electrophoretic mobility shift assay (EMSA), and key amino acid residues were identified through site-directed mutagenesis. The inhibitory effect of the cyclic peptide cyclosporin A (CsA) on Rgg-promoter binding was further assessed. The *rgg* gene (864 bp) encoding a 287-amino-acid protein (34.1 kDa) was successfully identified and expressed. Purified Rgg specifically bound to its own promoter region in a concentration-dependent manner. Mutations at conserved arginine residues R12 and R15 within the helix-turn-helix DNA-binding domain abolished promoter binding activity. Furthermore, CsA disturbed Rgg-promoter binding in a dose-dependent manner. This study provides the first in vitro characterization of an Rgg-family transcriptional regulator in fish-derived *S. parauberis*. The findings expand current understanding of Rgg-family regulators potentially associated with quorum sensing in aquatic streptococci and provide a preliminary basis for further investigation of quorum sensing-related regulatory interference strategies for controlling streptococcal diseases in aquaculture.

## 1. Introduction

*Streptococcus parauberis* is a major Gram-positive pathogen responsible for streptococcosis in a wide range of marine and freshwater fish species, including turbot, flounder, and channel catfish, leading to severe economic losses in global aquaculture industries [1,2,3,4,5]. In recent years, the emergence of novel serotypes and the rapid dissemination of multidrug resistance in *S. parauberis* have significantly reduced the effectiveness of conventional antibiotic-based treatments [6,7,8], raising serious concerns regarding environmental pollution, the dissemination of antimicrobial resistance in aquatic ecosystems, and the impact of antibiotic residues in aquaculture products on food safety [9].

Quorum sensing (QS) is a bacterial cell-to-cell communication mechanism that enables pathogens to coordinately regulate population-dependent behaviors, including virulence factor expression, biofilm formation, host colonization, and environmental adaptation [10,11,12,13]. Interference with QS systems has emerged as a promising alternative strategy for disease control, as it targets regulatory pathways linked to virulence without directly inhibiting bacterial growth and thus may reduce selective pressure for resistance development [14]. Compared with conventional approaches, vaccines are often limited by serotype specificity [15], while phage therapy can be constrained by narrow host range and stability [16]. In this context, targeting conserved QS regulators may provide a flexible and potentially broad-spectrum approach for modulating bacterial pathogenicity [17]. Notably, QS interference has been actively investigated in some aquaculture pathogens, particularly *Vibrio* species, with multiple studies demonstrating efficacy in reducing biofilm formation, virulence factor expression, and disease severity in fish [18].

Among QS systems in Gram-positive bacteria, the Rgg/small hydrophobic peptide (SHP) signaling pathway represents a recently characterized and widely distributed regulatory mechanism within the genus *Streptococcus* [19,20]. The Rgg-SHP system typically consists of an Rgg transcriptional regulator, which was first identified in *Streptococcus gordonii* that can regulates expression of glucosyltransferase [21] and its cognate SHP pheromone. Structurally, Rgg proteins possess a conserved N-terminal helix-turn-helix (HTH) DNA-binding domain and a C-terminal α-helical region responsible for peptide pheromone recognition [22]. Upon SHP internalization, Rgg regulators undergo conformational changes that modulate promoter binding and transcriptional regulation of target genes, thereby controlling diverse physiological processes such as biofilm development, capsule polysaccharide synthesis, carbon metabolism, and virulence-associated traits [23,24].

Extensive studies in human and veterinary streptococci, including *S. pyogenes*, *S. agalactiae*, *S. pneumoniae*, and *S. zooepidemicus*, have demonstrated that Rgg-SHP systems play critical roles in pathogenicity and host adaptation. Disruption of these systems results in attenuated virulence, altered biofilm formation, and impaired colonization capacity, suggesting that Rgg regulators may represent potential targets for further investigation in anti-virulence strategies [23,25,26,27]. Notably, quorum sensing inhibitors such as cyclic peptides and specialized microbial metabolites have been shown to effectively interfere with Rgg-SHP signaling, providing proof of concept for QS-targeted therapeutic development [28].

Despite increasing knowledge of Rgg-SHP systems in terrestrial and mammalian streptococci [14,15], their presence, molecular characteristics, and functional roles in fish-pathogenic *S. parauberis* remain poorly understood. In particular, experimental characterization of Rgg-family regulators and their susceptibility to quorum sensing interference compounds has not been systematically investigated.

In this study, we identified and characterized the Rgg transcriptional regulator from a fish-derived *S. parauberis* strain using bioinformatic, molecular, and biochemical approaches. We further investigated its promoter-binding activity, determined critical amino acid residues involved in promoter interaction through site-directed mutagenesis, and evaluated the inhibitory effect of the cyclic peptide cyclosporin A on Rgg function in vitro. This work provides fundamental insights into the potential regulatory role of an Rgg-family transcriptional regulator of *S. parauberis* and provides a preliminary basis for exploring quorum sensing-related regulatory mechanisms and potential intervention strategies in aquaculture.

## 2. Materials and Methods

### 2.1. Bacterial Strains, Plasmids, and Reagents

Fish-derived *S. parauberis* strain KRS02083 and plasmid pET-28a (+) were maintained in our laboratory. Strain KRS02083 was isolated from a diseased Japanese flounder (*Paralichthys olivaceus*). Genomic analysis (GenBank accession no. CP082783) indicated that it possesses multiple antimicrobial resistance genes and virulence factors, and it was chosen for this study as a representative isolate of *S. parauberis*. *Escherichia coli* DH5α and *E. coli* BL21 (DE3) were used for cloning and protein expression, respectively. Primers were synthesized by Sangon Biotech (Shanghai, China). Restriction enzymes, DNA polymerases, and molecular biology reagents were purchased from standard commercial suppliers.

### 2.2. Identification and Bioinformatic Analysis

The Rgg protein sequence of *S. dysgalactiae* (PDB ID: 4YV9) was used as a reference to identify homologous sequences in the complete genome of *S. parauberis* KRS02083 using TBLASTN (https://blast.ncbi.nlm.nih.gov/, accessed on 4 March 2026). The identified *rgg* gene (locus tag: K7G42_08775) was further analyzed for its theoretical molecular weight and isoelectric point using ExPASy (http://www.expasy.org). Homologous sequences were retrieved by BLASTp (https://blast.ncbi.nlm.nih.gov/, accessed on 4 March 2026), aligned using ClustalW v2.1, and a phylogenetic tree was constructed by the neighbor-joining (NJ) method in MEGA X v10.2.6. Conserved domains were predicted using NCBI CD-Search, and three-dimensional structural modeling was performed with Phyre2.2 to infer potential functional features [29]. The promoter region upstream of *rgg* was predicted using BPROM (http://www.softberry.com/). Primers (P1-Rgg F/P1-Rgg R; Table 1) for amplification of the predicted Rgg binding probe (P1) which covers the predicted promoter region were designed using Primer-BLAST (https://www.ncbi.nlm.nih.gov/tools/primer-blast/, accessed on 4 March 2026).

### 2.3. Cloning, Expression, and Purification

The *rgg* gene was amplified from the genomic DNA of *S. parauberis* strain KRS02083 using primers (S.p*rgg* F/S.p*rgg* R; Table 1) containing *Nde* I and *Xho* I restriction sites. The PCR product was ligated into the pET-28a (+) expression vector to generate pET-28a-*rgg*, which was subsequently transformed into *E. coli* BL21 (DE3) [30]. Transformants were cultured in LB medium supplemented with 100 μg/mL kanamycin at 37 °C with shaking at 200 rpm until the optical density at 600 nm reached approximately 0.5. Protein expression was induced with 0.8 mM isopropyl β-D-1-thiogalactopyranoside (IPTG), followed by overnight incubation at 25 °C and 140 rpm. A non-induced culture (without IPTG) was included as a negative control to assess basal expression levels. Cells were harvested by centrifugation and lysed by high-pressure homogenization. After centrifugation at 12,000 rpm for 20 min at 4 °C, the supernatant was collected and subjected to Ni-NTA affinity chromatography using an NGC™ chromatography system (Bio-Rad, Hercules, CA, USA). The recombinant Rgg protein was eluted with a linear imidazole gradient (50–500 mM). Protein purity was assessed by 12% SDS-PAGE, and purified fractions were stored at −80 °C for subsequent experiments [31].

### 2.4. Western Blot Analysis

Purified Rgg protein was separated by SDS-PAGE and transferred onto polyvinylidene fluoride (PVDF) membranes using a wet transfer system (Bio-Rad, USA). Membranes were blocked with 1% (*w*/*v*) skim milk at 4 °C overnight and then incubated with anti-His primary antibody (1:5000 dilution) for 2 h at room temperature with gentle shaking. After washing twice with TBST (10 min each), membranes were incubated with HRP-conjugated secondary antibody (1:10,000 dilution) for 1 h at room temperature. Protein bands were specifically detected using anti-His primary antibody in combination with HRP-conjugated secondary antibody. Following additional TBST washes, protein bands were visualized using an enhanced chemiluminescence (ECL) detection system (Clarity™ Western ECL Substrate, Bio-Rad, Hercules, CA, USA) [32].

### 2.5. Site-Directed Mutagenesis

Based on sequence alignment and structural analysis, two conserved arginine residues (R12 and R15) located within the predicted DNA-binding domain were selected for site-directed mutagenesis. Mutagenic primers (R12A F/R12A R and R15A F/R15A R) are listed in Table 1. Mutations were generated using a commercial mutagenesis kit (Fast Mutagenesis System Kit V2, TransGen, Beijing, China) according to the manufacturer’s instructions, with the wild-type pET-28a-*rgg* plasmid serving as the template. The wild-type plasmid was used as a positive control, while a no-template reaction was included as a negative control during PCR amplification. PCR products were treated with DMT enzyme at 37 °C for 1 h to remove parental plasmid DNA and transformed into chemically competent cells for screening [33]. Positive clones were verified by DNA sequencing, and non-mutated clones were used as negative controls. The mutant proteins were expressed and purified using the same procedures described for the wild-type Rgg protein.

### 2.6. Electrophoretic Mobility Shift Assay (EMSA)

DNA probes P1 and 16S rRNA were amplified from *S. parauberis* KRS02083 genomic DNA using primer pairs P1-Rgg F/R and 16S rRNA F/R, respectively. The forward primers were labeled at the 5′ end with HEX (Table 1). The P1 probe contained the predicted promoter region, whereas the 16S rRNA fragment served as a negative control (non-specific probe). Binding reactions (20 μL) were carried out in EMSA binding buffer containing 25 mM Tris-HCl (pH 7.5), 0.1 mM EDTA, 75 mM NaCl, 1 mM DTT, 10% glycerol, and 50 ng/μL poly(dI-dC). Each reaction included 100 nM labeled probe and increasing concentrations of purified Rgg protein (100, 300, and 500 nM). A probe-only sample without protein was included as a free probe control. The mixtures were incubated at room temperature for 30 min. Samples (8 μL) were separated on 5% native polyacrylamide gels in 0.5× TBE buffer at 4 °C and 100 V. Fluorescent signals were visualized using a Bio-Rad molecular imaging system [34]. The site-directed mutant Rgg proteins (R12A and R15A) were also subjected to EMSA under the same experimental conditions as the wild-type Rgg protein.

### 2.7. Cyclosporin A Interference Assay

Cyclosporin A (CsA; Sigma-Aldrich, St. Louis, MO, USA) was dissolved in dimethyl sulfoxide (DMSO). Purified Rgg protein (500 nM) was pre-incubated with increasing concentrations of CsA (0, 0.01, 0.1, 1, 5, 25 and 100 μM) in EMSA binding buffer (25 mM Tris-HCl, pH 7.5, 0.1 mM EDTA, 75 mM NaCl, 1 mM DTT, and 10% glycerol) supplemented with 50 ng/μL poly(dI-dC) on ice for 30 min. An equivalent volume of DMSO was included as a solvent control. Subsequently, the HEX-labeled P1 probe (100 nM) was added, and the reaction mixture was incubated at room temperature for an additional 30 min. A probe-only sample without protein was included as a free probe control. Samples were separated on 5% native polyacrylamide gels in 0.5× TBE buffer at 4 °C and 100 V. The EMSA was visualized by a Bio-Rad molecular imaging system [34].

## 3. Results

### 3.1. Bioinformatic Analysis of Rgg

**Figure 1:**
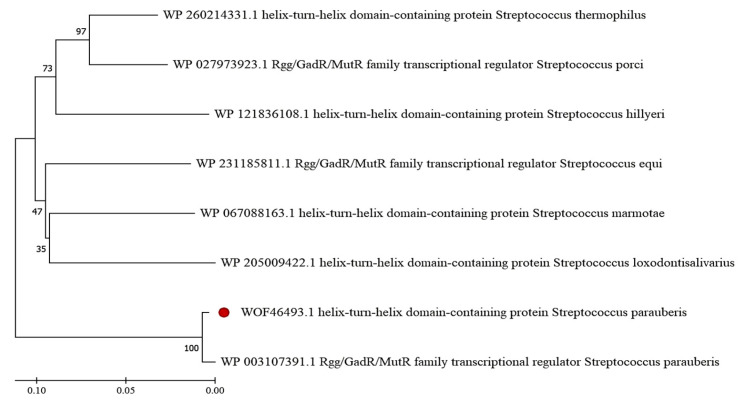
Phylogenetic analysis of *S. parauberis* Rgg. The numbers at the nodes represent the bootstrap values (out of 1000 bootstrap replications); the scale bar represents a 5% sequence difference, the shorter the ruler, the closer the kinship. The red circle indicates the *S. parauberis* Rgg protein analyzed in this study.

**Figure 2:**
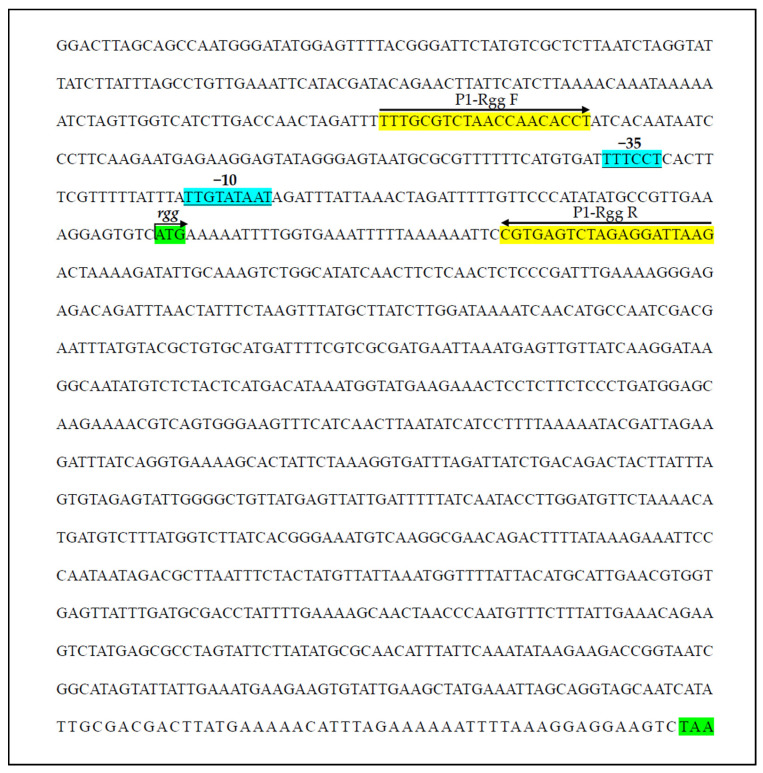
Predicted promoter elements of *rgg* and the design of the Rgg-binding probe (P1). The −10 and −35 boxes are indicated in blue with underlining. Primers used for amplification of the P1 probe are shown in yellow with arrowheads. The start and stop codons of the *rgg* open reading frame (ORF) are highlighted in green. The arrow above indicates the direction of transcription.

The *rgg* gene identified in *S*. *parauberis* strain KRS02083 was 864 bp in length and encoded a 287-amino-acid protein. The predicted molecular weight and theoretical isoelectric point were 34.1 kDa and 7.68, respectively. Sequence alignment revealed high conservation of Rgg within *S. parauberis* (98.95% identity) and substantial identity (>79%) with homologs from *S. thermophilus*, *S. porci*, and *S. equi*, indicating strong conservation of Rgg proteins across the genus. Phylogenetic analysis grouped the eight selected Rgg homologs into two distinct clades, with *S. parauberis* Rgg forming a separate branch (Figure 1). Conserved domain analysis identified an N-terminal helix-turn-helix (HTH) XRE-type DNA-binding domain (amino acids 5–61) and a C-terminal Rgg regulatory domain (amino acids 63–285). Structural modeling using Phyre2.2 predicted that 267 residues (93% of the sequence) could be modeled with 100% confidence based on the crystal structure of the *S. dysgalactiae* SHP pheromone receptor Rgg2 (PDB: 4YV9), supporting the presence of a canonical N-terminal DNA-binding domain and a C-terminal SHP-binding domain. The predicted promoter elements of *rgg* and the designed P1 probe are shown in Figure 2.

### 3.2. Expression, Purification, and Identification of Rgg

**Figure 3:**
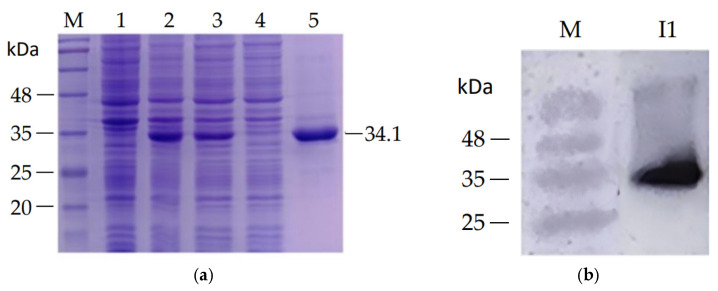
Expression and purification of recombinant Rgg protein (**a**) and its identification by Western blot analysis (**b**). M: protein marker; lane 1: uninduced sample; lane 2: induced whole-cell lysate; lane 3: supernatant after sonication; lane 4: flow-through fraction; lane 5: purified Rgg protein; I1: immunoreactive band corresponding to His-tagged Rgg.

The recombinant plasmid pET-28a-*rgg* was transformed into *E. coli* BL21 (DE3) for heterologous expression. Following IPTG induction and Ni-NTA affinity purification, a protein band corresponding to the expected molecular weight (34.1 kDa) was observed by SDS-PAGE (Figure 3a). Western blot analysis using an anti-His monoclonal antibody detected a single immunoreactive band at the same molecular weight, confirming that the purified protein was the target His-tagged Rgg (Figure 3b).

### 3.3. Analysis of Rgg Binding to the Promoter Region by EMSA

**Figure 4:**
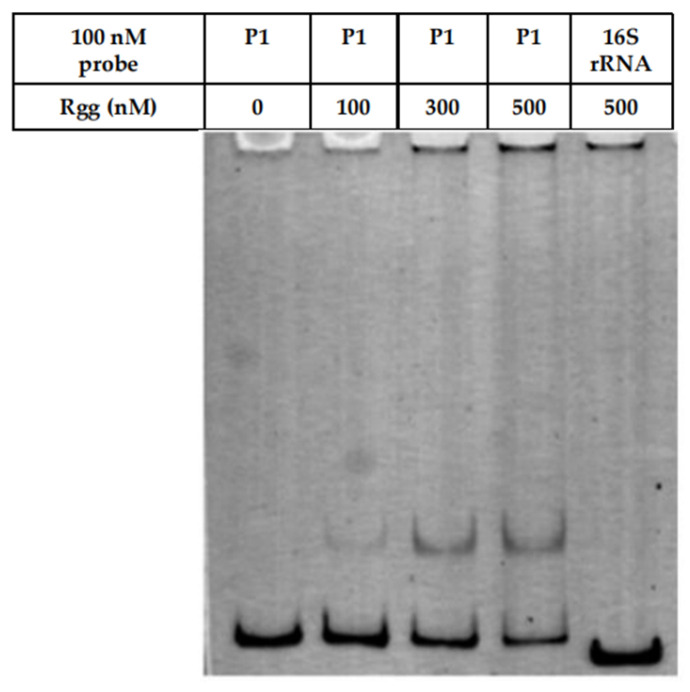
EMSA analysis of the binding of wild-type Rgg protein to the predicted promoter region. P1: specifc probe; 16S rRNA: non-specifc probe; 0, 100, 300, and 500: Rgg protein concentrations of 0, 100, 300, and 500 nM, respectively.

To evaluate the DNA-binding activity of Rgg, EMSA was performed using a probe P1, which encompassing the predicted *rgg* promoter region. As shown in Figure 4, the binding of Rgg to the P1 probe increased progressively with rising protein concentrations (100, 300, and 500 nM), whereas no binding was observed with the 16S rRNA negative control probe, indicating the binding specificity of Rgg.

### 3.4. Identification of Key Amino Acid Residues Involved in Promoter Binding

**Figure 5:**
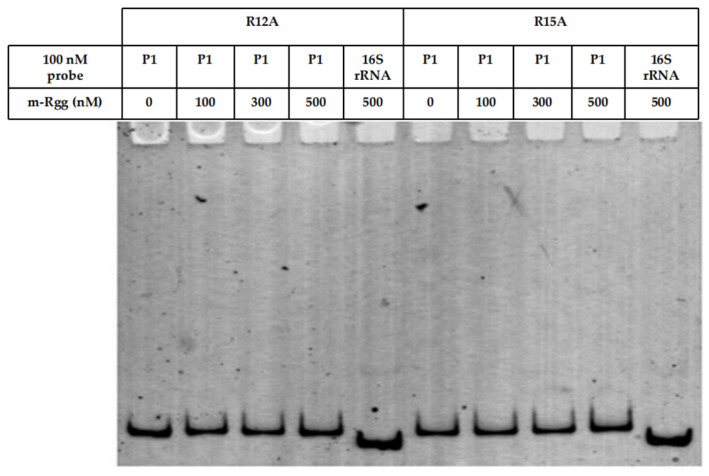
EMSA analysis of the promoter-binding ability of site-directed mutant Rgg proteins (R12A and R15A). P1: specifc probe; 16S rRNA: non-specifc probe; 0, 100, 300, and 500: mutant Rgg protein concentrations of 0, 100, 300, and 500 nM, respectively.

To identify the key amino acid residues involved in promoter binding, conserved residues were selected for site-directed mutagenesis based on sequence alignment of homologous Rgg proteins. EMSA results showed that substitution of arginine at positions 12 and 15 abolished the DNA-binding activity of Rgg, as no detectable interaction with the probe was observed (Figure 5). These findings indicate that R12 and R15 are essential for Rgg-DNA interaction.

### 3.5. Effect of CsA on Rgg Function

**Figure 6:**
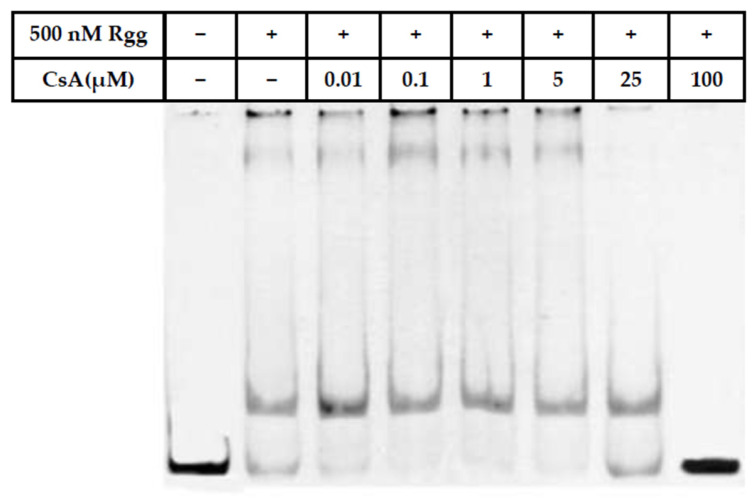
EMSA analysis of the effect of CsA on the promoter-binding activity of Rgg.

To assess the impact of CsA on Rgg activity, purified Rgg protein was incubated with increasing concentrations of CsA prior to EMSA analysis. As shown in Figure 6, within the range of 0.01–100 μM, Rgg-P1 probe binding initially increased and subsequently decreased with rising CsA concentrations. At 100 μM CsA, Rgg completely lost its ability to bind the promoter probe P1.

## 4. Discussion

Quorum sensing (QS) systems are increasingly recognized as pivotal regulators of virulence, environmental adaptation, and biofilm formation in Gram-positive pathogens, particularly within the genus *Streptococcus* [35]. However, in *S. parauberis*, a major etiological agent of streptococcosis in marine and freshwater fish, the molecular mechanisms underlying QS regulation remain insufficiently characterized. Based on the conserved roles of Rgg-family regulators in other streptococci [19], we hypothesized that the Rgg protein in *S. parauberis* functions as a quorum sensing-associated transcriptional regulator capable of binding promoter regions and that its activity may be modulated by small-molecule compounds. In the present study, we experimentally identified and biochemically characterized an Rgg-family transcriptional regulator from a fish-derived *S. parauberis* and demonstrated its specific DNA-binding activity, the essential role of conserved N-terminal residues in promoter recognition, and its susceptibility to modulate by cyclosporin A (CsA). These findings provide basic insights into QS-associated regulatory mechanisms in this important aquaculture pathogen. In addition, Gram-negative pathogens in aquaculture such as *Vibrio harveyi* and *Aeromonas hydrophila*, quorum sensing plays a central role in controlling biofilm formation, virulence, and environmental persistence [36,37]. Although the molecular mechanisms differ between Gram-positive and Gram-negative bacteria, the overarching principle of cell-density-dependent regulation of pathogenic traits is conserved [38]. In this context, our findings provide preliminary biochemical evidence supporting a potential regulatory role of Rgg in *S. parauberis*, although its broader regulon and physiological functions remain to be determined.

### 4.1. Functional Conservation and Structural Features of S. parauberis Rgg

Bioinformatic analyses revealed that the *rgg* gene of *S. parauberis* encodes a 287-amino-acid protein containing a canonical N-terminal helix-turn-helix (HTH) XRE-type DNA-binding domain and a C-terminal regulatory domain. The high sequence identity with Rgg homologs from other *Streptococcus* species and the strong structural similarity predicted by homology modeling support the classification of this protein within the classical Rgg family [39]. Rgg regulators are widely distributed in streptococci and typically function as transcription factors responding to short hydrophobic peptides (SHPs), thereby modulating gene expression in a cell-density-dependent manner [40].

The predicted three-dimensional model based on the crystal structure of *S. dysgalactiae* Rgg2 suggests conservation of the overall domain architecture, including the N-terminal DNA-binding domain and a C-terminal SHP pheromone-binding domain. Such structural conservation implies a similar mechanistic framework in *S. parauberis*, where SHP ligand binding at the C-terminus may induce conformational changes that alter DNA-binding affinity or promoter specificity [22,41]. The phylogenetic clustering of *S. parauberis* Rgg into a distinct branch may indicate species-specific regulatory adaptations, possibly reflecting ecological niche specialization in aquatic environments [42].

### 4.2. Specific DNA Binding and Autoregulatory Potential

EMSA results demonstrated that recombinant Rgg specifically binds to the predicted promoter region upstream of *rgg*, while no interaction was observed with a non-specific 16S rRNA probe. The concentration-dependent shift further confirms direct protein-DNA interaction. These findings strongly support the hypothesis that Rgg likely functions as a transcriptional regulator in *S. parauberis* [43].

Notably, the use of a promoter fragment located upstream of the *rgg* suggests that Rgg may participate in autoregulation. Autoregulatory circuits are common among QS regulators and often contribute to signal amplification, rapid response to environmental cues, and stabilization of gene expression states [44]. In other streptococci, Rgg proteins have been shown to act either as transcriptional activators or repressors depending on promoter context and peptide ligand availability [45]. Although the present study did not directly assess transcriptional outcomes in vivo, the observed promoter binding provides a mechanistic basis for potential autoregulatory or feedback control loops in *S. parauberis*. However, it should be emphasized that binding to the autoregulatory promoter alone does not establish the full regulatory scope of Rgg within the quorum sensing network, nor does it demonstrate its involvement in controlling downstream target genes. Therefore, the role of Rgg as a global regulator remains to be further validated through genome-wide and in vivo approaches [46].

### 4.3. Critical Role of Conserved Arginine Residues in DNA Recognition

Site-directed mutagenesis revealed that substitution of arginine residues at positions 12 and 15 completely abolished DNA-binding activity. These residues are located within the predicted HTH motif, a structural element known to mediate major groove recognition of target DNA sequences [47]. Arginine residues frequently contribute to DNA binding through electrostatic interactions with the negatively charged phosphate backbone and formation of hydrogen bonds with specific base pairs [48].

The complete loss of promoter binding upon R12A or R15A substitution highlights their indispensable role in stabilizing the Rgg-DNA complex. This observation is consistent with the structural paradigm of XRE-family transcription factors, in which positively charged residues within the recognition helix are crucial for sequence-specific binding [49]. These data provide experimental validation of the predicted DNA-binding domain and clarify the molecular determinants of promoter interaction in *S. parauberis* Rgg.

### 4.4. Modulation of Rgg Activity by CsA

An important finding of this study is that CsA interferes with Rgg-DNA interaction in a concentration-dependent manner. Interestingly, low concentrations of CsA initially enhanced DNA-binding activity, whereas higher concentrations progressively reduced binding, culminating in complete inhibition at 100 μM. This biphasic effect may reflect complex allosteric modulation of Rgg structure [50]. However, the requirement for relatively high concentrations to achieve complete inhibition suggests that these effects may not reflect physiologically or environmentally relevant conditions in aquaculture systems [51].

CsA is a cyclic peptide with well-established immunosuppressive activity in eukaryotic systems, but it has also been reported to interact with bacterial regulatory proteins in specific contexts [41]. One possible explanation is that low concentrations of CsA stabilize a conformation of Rgg favorable for DNA interaction, whereas higher concentrations disrupt the structural integrity of the DNA-binding domain or interfere with protein oligomerization. Alternatively, CsA may mimic peptide ligands that interact with the C-terminal regulatory domain, thereby altering the equilibrium between active and inactive conformations [25,41].

From an application perspective, these findings provide preliminary evidence that small molecules can modulate Rgg-DNA interactions, supporting the feasibility of targeting regulatory pathways through quorum sensing interference (QSI) related strategies [25]. However, given the well-known immunosuppressive properties of CsA, its direct application in aquaculture is unlikely to be feasible or appropriate, as it may compromise host immune function. Therefore, CsA should be considered primarily as a proof-of-concept molecule for identifying Rgg-targeting compounds rather than a practical therapeutic agent. Future work should focus on identifying more potent, non-immunosuppressive compounds or structural analogs with improved specificity and lower effective concentrations, which may offer greater translational potential for disease control in aquaculture systems [52].

### 4.5. Limitations and Future Perspectives

Several limitations should be acknowledged. First, the regulatory targets of Rgg beyond its own promoter were not identified in this study. Genome-wide approaches, such as electrophoretic mobility shift coupled with sequencing (EMSA-seq), chromatin immunoprecipitation sequencing (ChIP-seq), or transcriptomic profiling of *rgg* mutants, would clarify the broader regulon. Second, in vivo functional studies using gene knockout or complementation strains are needed to directly link Rgg to virulence phenotypes and disease outcomes in fish. Third, the molecular mechanism underlying CsA-mediated modulation requires structural and biophysical investigation to determine whether direct binding occurs and to define interaction sites.

Future research should therefore focus on (i) identifying downstream genes controlled by Rgg, (ii) elucidating peptide ligands or signaling molecules involved in this regulatory pathway, and (iii) evaluating quorum sensing interference strategies under aquaculture-relevant conditions.

Overall, the present study provides the first in vitro biochemical characterization of an Rgg-family regulator in fish-derived *S. parauberis*, providing initial insights into quorum sensing-associated regulatory components in aquatic streptococci.

From a biological perspective, the ability of Rgg to specifically bind its promoter and to be modulated by a small molecule suggests that QS-associated regulatory pathways may be involved in the coordination of gene expression in this pathogen, although their direct roles in virulence and host interaction remain to be demonstrated.

These findings therefore establish a foundation for future studies aimed at linking Rgg-mediated regulation to phenotypic traits relevant to infection, such as biofilm formation, colonization, and pathogenicity in aquaculture hosts.

## 5. Conclusions

In summary, this study provides the first experimental evidence that the Rgg protein of fish-derived *S. parauberis* functions as a sequence-specific DNA-binding regulator, with essential arginine residues in the N-terminal HTH domain mediating promoter recognition. However, its broader regulatory role within quorum sensing networks and its downstream targets remain to be elucidated. The observed impact on DNA-binding activity by CsA further supports the feasibility of suggesting that Rgg may represent a potential target for quorum sensing-related regulatory interference, although further studies are required to identify biologically relevant and safe modulators. These findings expand our understanding of QS-related regulation in aquatic streptococci and offer a conceptual framework for future studies exploring alternative intervention strategies against streptococcosis in aquaculture.

## Figures and Tables

**Table 1:** Primers used for gene cloning, mutagenesis, and probe preparation in this study.

Primer	Sequence (5′-3′)	Purpose
S.p*rgg* F	GGGAATTCCATATGAAAAATTTTGGTGAAATTTT	Cloning of *rgg*
S.p*rgg* R	CCGCTCGAGTTAGACTTCCTCCTTTAAAA	
R12A F	AATTTTTAAAAAATTCGCTGAGTCTAGAGGATTAA	Site-directed mutagenesis
R12A R	GCGAATTTTTTAAAAATTTCACCAAAATTTTTCAT	
R15A F	AAAATTCCGTGAGTCTGCAGGATTAAGACTAAAAG	Site-directed mutagenesis
R15A R	GCAGACTCACGGAATTTTTTAAAAATTTCACCAAA	
16S rRNA F	TGCCTGCAGGTCGACGATACCCCTATTGTTAGTTGCCAT	Non-specific probe amplification
16S rRNA R	GTTGCAGCCTACAATCCGAAC	
P1-Rgg F	TGCCTGCAGGTCGACGATTTTGCGTCTAACCAACACCT	EMSA probe (P1) amplification
P1-Rgg R	CTTAATCCTCTAGACTCACG	
HEX F	TGCCTGCAGGTCGACGAT	5′-HEX labeling adapter

The underlines represent the restriction sites. The red font indicates the mutation sites.

## Data Availability

The original contributions presented in this study are included in the article. The genome sequence of *Streptococcus parauberis* strain KRS02083 used for bioinformatic analysis is publicly available in the NCBI GenBank database under accession number CP082783. Further inquiries can be directed to the corresponding author.

## Abbreviations

The following abbreviations are used in this manuscript:

QS	Quorum sensing
QSI	Quorum sensing interference
EMSA	Electrophoretic mobility shift assay
CsA	Cyclosporin A
SHP	Small hydrophobic peptide
HTH	Helix-turn-helix
IPTG	isopropyl β-D-1-thiogalactopyranoside
SDS-PAGE	Sodium dodecyl sulfate polyacrylamide gel electrophoresis
PVDF	Polyvinylidene fluoride
DMSO	Dimethyl sulfoxide
ChIP-seq	Chromatin immunoprecipitation sequencing
